# Identification of differential co-expressed gene networks in early rheumatoid arthritis achieving sustained drug-free remission after treatment with a tocilizumab-based or methotrexate-based strategy

**DOI:** 10.1186/s13075-017-1378-x

**Published:** 2017-07-20

**Authors:** Xavier M. Teitsma, Johannes W. G. Jacobs, Michal Mokry, Michelle E. A. Borm, Attila Pethö-Schramm, Jacob M. van Laar, Johannes W. J. Bijlsma, Floris P. J. Lafeber

**Affiliations:** 1 Department of Rheumatology & Clinical Immunology, University Medical Center Utrecht, Heidelberglaan 100, 3584 CX Utrecht, Netherlands; 2 Epigenomics Facility, University Medical Center Utrecht, Heidelberglaan 100, 3584 CX Utrecht, Netherlands; 3 Division of Paediatrics, University Medical Center Utrecht, Heidelberglaan 100, 3584 CX Utrecht, Netherlands; 4 Roche Nederland BV, Beneluxlaan 2a, 3446 GR Woerden, Netherlands; 5 F Hoffmann-La Roche, Grenzacherstrasse 124, 4070 CH Basel, Switzerland

**Keywords:** Rheumatoid arthritis, Tocilizumab, Methotrexate, Drug-free remission, Weighted gene co-expression network analysis

## Abstract

**Background:**

Methotrexate is endorsed to be used as first-line treatment in rheumatoid arthritis (RA). However, a large proportion of patients need additional treatment with a biological disease-modifying anti-rheumatic drug (DMARD) to adequately suppress their disease activity. A better understanding of genotypes could help to distinguish between patients with different pathogenic mechanisms. The aim of this study was therefore to identify networks of genes within DMARD-naive early RA patients associated with achieving sustained drug-free remission (sDFR) after initiating tocilizumab plus methotrexate, tocilizumab, or methotrexate therapy.

**Methods:**

Samples were used from 60 patients from the U-Act-Early study who received tocilizumab plus methotrexate, tocilizumab, or methotrexate therapy, and who achieved sDFR (≥3 months in drug-free remission until the end of the study, *n* = 37) after therapy was tapered and subsequently stopped, or who were not able to discontinue the therapy as controls (*n* = 23). Whole blood samples were collected and ribonucleic acid (RNA) was isolated from positive cluster of differentiation 4 (CD4^+^) and CD14^+^ cells and analysed using high-throughput sequencing. Weighted gene co-expression network analyses were performed to identify clusters (i.e. modules) of differently expressed genes associated with achieving sDFR and which were subsequently used for pathway analyses.

**Results:**

Network analyses within CD4^+^ cells identified two significant modules in the tocilizumab plus methotrexate arm and four modules in the tocilizumab and methotrexate arms, respectively (*p* ≤ 0.039). Important pathways in the module best correlating with achieving sDFR were in the tocilizumab plus methotrexate arm related to processes involved with transcription and translation; in the tocilizumab arm, pathways were related to migration of white blood cells and G-protein coupled receptors, and in the methotrexate arm pathways were involved with the response to a bacterial or biotic (i.e. biological material)-related stimulus. No relevant networks could be identified in the sequenced CD14^+^ cells.

**Conclusions:**

Within networks of co-expressed genes, several pathways were found related to achieving sDFR after initiating therapy with tocilizumab, methotrexate, or the combination. Between the three strategy arms, we identified different networks of predisposing genes which indicates that specific gene expression profiles, depending on the treatment strategy chosen, are associated with a higher chance of achieving sDFR.

**Trial registration:**

Clinicaltrials.gov, NCT01034137. Registered on 16 December 2009.

**Electronic supplementary material:**

The online version of this article (doi:10.1186/s13075-017-1378-x) contains supplementary material, which is available to authorized users.

## Background

Rheumatoid arthritis (RA) is an autoimmune disease characterized by synovial tissue inflammation of the peripheral joints, often followed by destruction of those affected joints [1, 2]. Although the cause of RA is not fully understood, it is recognized to be a multifactorial disease involving both environmental and genetic factors that influence the susceptibility and severity of the disease [3–5]. Approximately 50% of the risk for developing RA is attributable to genetic predisposing factors. Previous studies have shown that, for example, the protein tyrosine phosphatase, non-receptor type 22 (PTPN22) and peptidyl arginine deiminase, type 14 (PADI4) genes and various human leucocyte antigen (HLA) class II alleles are associated with an increased risk [4, 6–8]. Furthermore, the heterogeneity of RA is demonstrated by the variability in the presence of autoantibodies, the diverse clinical disease manifestations and different grades of responses to disease-modifying anti-rheumatic drugs (DMARDs). Adequately targeting the disease from the start is essential to preserve physical function and ensure long-term beneficial outcome, especially in early RA [9]. A better understanding of gene expression profiles could help to distinguish between patients with different pathogenic mechanisms and could lead to improved clinical outcomes when more individualised therapy is initiated from the start. An anchor drug in the treatment of RA is methotrexate, one effect of which is inhibition of dihydrofolate reductase that is not only important for cell proliferation and cell growth but also for inducing protein synthesis [10]. The various mechanisms of methotrexate involved in the treatment of RA still have not yet been fully elucidated, but it is believed that an anti-inflammatory effect also plays a large role as in vitro and in vivo studies have demonstrated previously [11]. Although methotrexate is recommended as an initial treatment strategy [12], a large number of patients needs additional treatment with a biological DMARD or withdraw from this therapy because of inefficacy or adverse effects [13–15]. Tocilizumab is a humanized monoclonal antibody inhibiting interleukin (IL)-6 signalling by blocking the binding of IL-6 to its receptor, and has been proven in randomized controlled trials to be effective across different RA populations in reducing disease activity and inhibiting the progression of joint damage [16–28]. IL-6 is a pro-inflammatory cytokine that is secreted by multiple cell types (e.g. T cells, B cells, monocytes, and osteoblasts) and stimulates the immune response and osteoclast formation [29, 30]. In the present study, we aimed to identify biological networks and signature protein coding genes that are associated with achieving sustained drug-free remission (sDFR) after initiating treatment with tocilizumab, methotrexate, or the combination of both, by performing whole transcriptome ribonucleic acid sequencing (RNA-seq) of positive cluster of differentiation 4 (CD4^+^) and CD14^+^ cells obtained from DMARD-naive early RA patients. Additionally, we were interested whether pathways related to achieving sDFR were treatment dependent by investigating gene expression profiles between the tocilizumab and methotrexate arms, or whether sDFR was dependent on general predisposing factors present within all strategy arms. As genotypes differ between RA patients, identification of biological pathways associated with achieving sDFR could enable application of more personalized treatment strategies which, in those who are newly diagnosed, would result in better disease activity control and prevention of disease progression.

## Methods

### Design

Data was used from the 2-year, multicentre, double-blind, placebo-controlled, randomized U-Act-Early trial (ClinicalTrials.gov identifier: NCT01034137) in which DMARD-naive patients with early RA were treated-to-target with tocilizumab plus methotrexate, or tocilizumab, or methotrexate therapy. The study design and details have been described previously [21]. Briefly, 317 patients with active RA (disease activity score assessing 28 joints (DAS28) >2.6) were randomized (1:1:1) to initiate tocilizumab (8 mg/kg), step-up methotrexate (starting dose 10 mg/week), or tocilizumab plus methotrexate therapy and were treated until the treatment target (sustained remission; defined as a DAS28 < 2.6 and a maximum swollen joint count of ≤4 for ≥24 weeks) was achieved. If remission was not reached, hydroxychloroquine (200 mg twice per day) was added to the regimen (initial treatment strategy) and was discontinued 12 weeks thereafter if the target still was not achieved. Patients who did not achieve remission following the initial treatment strategy switched to a subsequent treatment regimen; patients who started with tocilizumab or methotrexate therapy then switched to combination therapy with tocilizumab plus methotrexate, and those who had initiated this treatment strategy switched to the standard of care (i.e. methotrexate combined with a tumour necrosis factor inhibitor). When the primary outcome, sustained remission, was achieved, medication was tapered and discontinued if remission was maintained. Methotrexate was first tapered at 5-mg/4-week steps until 10 mg and then discontinued; thereafter tocilizumab was tapered to 4 mg/kg and finally stopped after 3 months. For the present study, we used data from those achieving sDFR (defined as being drug-free for ≥3 months and maintaining this state until the end of the 2-year study period where one visit of low-disease activity (DAS28 < 3.2) was allowed) in the three treatment strategies. As controls, we selected patients within each treatment who never achieved a drug-free status at any time point during the whole study period, although they could have achieved sustained remission and subsequently were tapering medication.

**Fig. 1:**
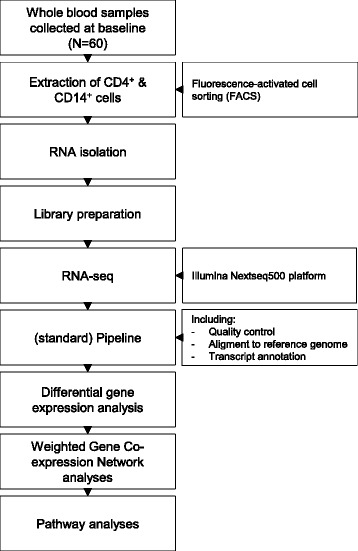
A Flowchart of the study. Whole blood samples were collected from 60 patients and thereafter CD4^+^ and CD14^+^ cells were extracted using fluorescence-activated cell sorting (*FACS*). Then RNA was isolated from these cells and, via reverse transcription, the library was prepared and sequenced (*RNA-seq*). Thereafter, a (standard) pipeline was applied for quality control and processing of the reads; read counts were used for detecting differentially expressed genes (DEGs) and these were then used for detecting networks of co-expressed genes. Pathway analyses in the Gene Ontology (GO) and Kyoto Encyclopedia of Genes and Genomes (KEGG) databases were performed in the most important networks

Before patients received their first dose of medication, whole blood samples were collected and CD4^+^ plus CD14^+^ cells were extracted using fluorescence-activated cell sorting (FACS). Thereafter, total RNA was isolated from these cells using the RNeasy® Mini Kit (Qiagen, Netherlands) following the manufacturer’s recommendations. The quantity of the isolated RNA was assessed with ND1000/ND2000 NanoDrop® and the quality was assessed with the Agilent 2100 Bioanalyzer® (Agilent Technologies, Germany). The mean (standard deviation (SD)) RNA Integrity Number (RIN) of the samples was 9.3 (0.3) with a mean (SD) RNA concentration of 220 (76) ng/µl. Thereafter, the RNA tubes were stored at −80 °C. For the RNA-seq, libraries were constructed using the NEXTflex™ Rapid RNA-Seq kit (Bio Scientific) and mRNA was selected using NEXTflex™ poly-A tail following the manufacturer’s recommendations. Thereafter, the library was ready and sent to the Utrecht Sequencing Facility to be sequenced on the Illumina Nextseq500 platform using a single-end 75-base pair high-output run. After the library was sequenced, a standard pipeline was used that includes a quality control, processing of the reads, alignment to a reference genome, transcript annotation, and finally an estimation of the read counts of the transcripts. A flowchart of the study is presented in Fig. 1.

### Statistical analyses

Principal component analysis (PCA) of the raw read counts was performed to identify possible confounders for the level of expression. Thereafter, the DESeq2 package was used to test for differential gene expression by use of negative binomial generalized linear models [31]. The model was corrected for gender and C-reactive protein (CRP) levels as they had been identified as confounders in the PCA (data not shown). The weighted gene co-expression network analysis (WGCNA) package was then used to construct networks of co-expressed genes (i.e. modules) of the 1000 most differentially expressed genes (DEGs) among the three treatment arms [32]. A continuous (range −1,1) adjacency matrix was constructed for all pairs of genes using Pearson’s correlation coefficient (PCC). Subsequently, the correlation matrix was transformed into connection strengths using a soft threshold to obtain a scale-free network whereas the threshold power, based on the explained variance, was determined separately for each treatment arm. The connection strengths were then used to calculate the topological overlap matrix, which measures the interconnectivity of pairs of genes. Modules of correlated genes were identified using hierarchical average clustering, which groups genes with similar expression patterns, and modules that were highly correlated (PCC ≥0.80) were merged. Each module is labelled by a colour and the minimum number of genes per module was set at 20, except for the tocilizumab plus methotrexate arm (CD14^+^ cells) and tocilizumab arm (CD4^+^ cells) where the threshold was set at 10 to create sufficient numbers of modules. Genes not assigned to any of the modules were housed in the grey module and disregarded. After the modules had been identified and merged, the module eigengene (*E*) was calculated (range 0–1; with “1” indicating highest *E*), which can be considered as the first principal component of the expression matrix and thus representing the average gene expression profile within the module. The module that correlated best with achieving sDFR (no = 0, yes = 1) was considered the most relevant and was used for further pathway analysis. Additionally, the correlation between clinical traits and the expression profile was also analysed to determine if other factors besides achieving sDFR were important in the module of interest. Gene Ontology (GO) and Kyoto Encyclopedia of Genes and Genomes (KEGG) pathway analyses were performed using the GOseq package, which accounts for gene length bias using a probability weighting function [33]. *P* values were corrected for multiple testing when performing pathway analysis (Benjamini-Hochberg correction) [34]. VisANT 5.0 software was used to visualize the connectivity between genes in the module of interest and to identify signature genes that are considered to play an important role in the biological processes as they are characterized by a high connectivity with other genes [35, 36]. Statistical significance was determined at *P* < 0.05 (two tailed) and network analyses were performed using the statistical program R version 3.3.1.

## Results

**Fig. 2:**
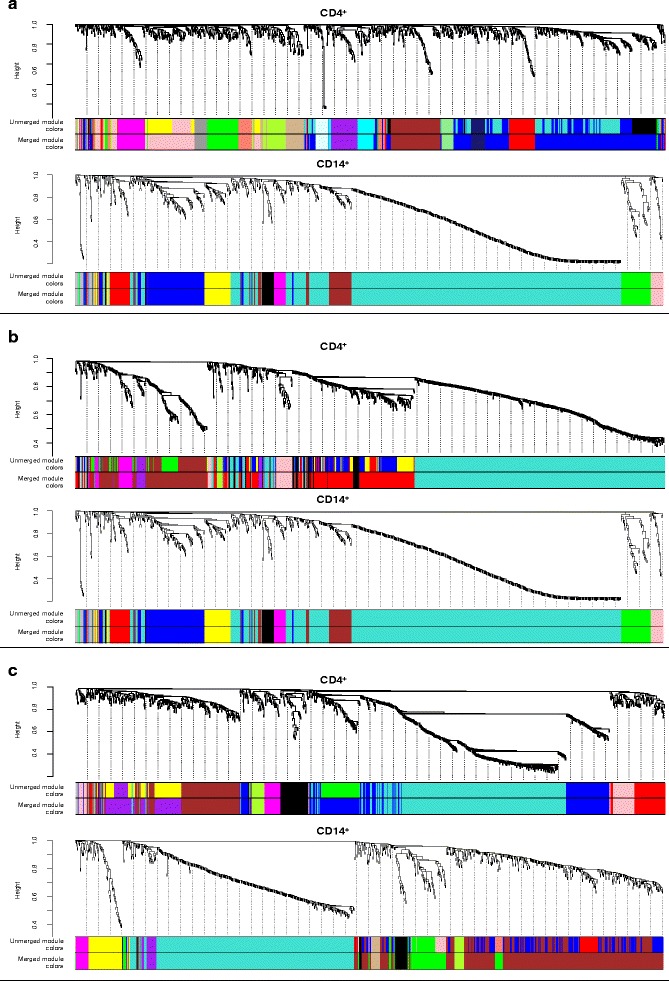
Hierarchical cluster dendrograms of the differently expressed genes included in the network analysis within the (**a**) tocilizumab plus methotrexate, (**b**) tocilizumab, and (**c**) methotrexate strategy arms. Each line represents an individual gene and the branches correspond to modules of co-expressed genes, which are labelled by colours

In total, 60 individual patient samples (tocilizumab plus methotrexate, 19 (*n* = 14 achieved sDFR, *n* = 5 controls); tocilizumab, 24 (*n* = 13 achieved sDFR, *n* = 11 controls); methotrexate, 17 (*n* = 10 achieved sDFR, *n* = 7 controls)) were included in the present analysis. The mean (SD) age of all patients was 53 (14) years with a median (interquartile range (IQR)) symptom duration of 23 (18–40) days; 60% were rheumatoid factor positive and 60% anti-cyclic citrullinated peptide positive. The majority were female (68%) and patients had at baseline a mean (SD) DAS28 of 4.9 (1.1); median (IQR) CRP level was 9 (3–19) mg/L and erythrocyte sedimentation rate 20 (12–32) mm/h. No significant differences in clinical characteristics were found between those achieving sDFR and the controls within the strategy arms (*p* ≥ 0.07, Additional file 1: Table S1). For each sample, the expression of approximately 20,000 protein coding genes was measured in both CD4^+^ and CD14^+^ cells and normalized read counts were determined for each gene. Hierarchical cluster analyses of the relevant DEGs are shown in Fig. 2. In the sequenced CD4^+^ cell population, weighted network analyses identified two, four, and four modules after merging that were significantly correlated with achieving sDFR in the tocilizumab plus methotrexate, tocilizumab, and methotrexate strategy arms, respectively (Table 1). In the tocilizumab plus methotrexate arm, the salmon module was found to be most significantly related to sDFR (PCC 0.58, *p* = 0.009); in the tocilizumab arm it was the purple module (PCC 0.52, *p* = 0.009), and in the methotrexate arm the black module (PCC 0.60, *p* = 0.010). The number of genes included in the modules is 40, 26, and 49, respectively (Additional file 2: Table S2). When performing network analyses within the CD14^+^ cells, no modules were found that were significantly correlated with achieving sDFR in the tocilizumab plus methotrexate and methotrexate arms (Additional file 3: Table S3). In the tocilizumab arm, only one module (pink) was significantly correlated (PCC 0.41, *p* = 0.049). However, within this module, symptom duration was an important contributing trait as it was highly correlated (PCC 0.93, *p* < 0.001) with the average expression profile (*E*). Because the correlation between the pink module and achieving sDFR was low (<0.50) and symptom duration was a more contributing factor to the gene expression in this module, it was disregarded for further analyses. Pathway analyses were therefore only performed for the modules found relevant within the CD4^+^ cells of the three treatment arms.

**Table 1:**
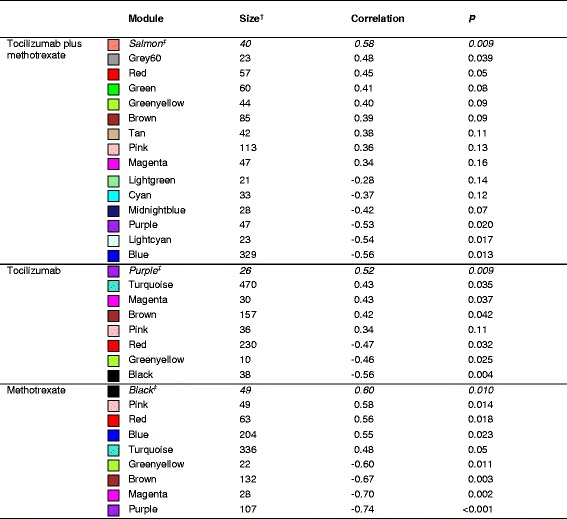
Correlations and corresponding *P* values between modules and achieving sustained drug-free remission within sequenced CD4^+^ cells

The module eigengene, which is the first principal component of the expression matrix within the module of interest, was considered as the average gene expression profile and was used to test the correlation between each module and achieving sustained drug-free remission

^‡^The modules with the highest significant correlation (shown in italic) were considered most relevant and were selected for further functional pathway analysis

^†^The minimal number of genes per module was set at 20, except for the tocilizumab arm (*n* = 10)

### GO and KEGG pathway analysis

**Fig. 3:**
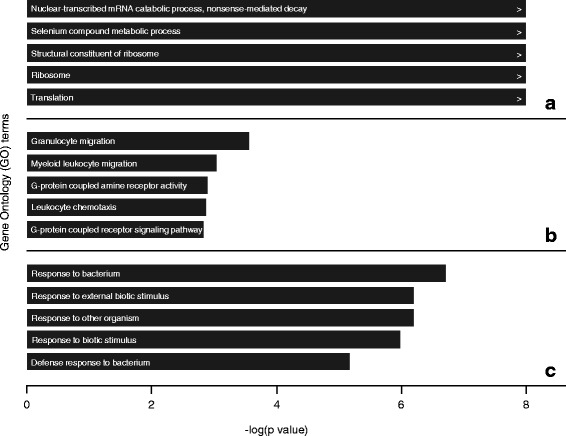
The top five most significantly overrepresented gene ontology (*GO*) terms within the (**a**) tocilizumab plus methotrexate, (**b**) tocilizumab, and (**c**) methotrexate strategy arms. GO terms with >1 DEGs were included in this overview

In the salmon module (tocilizumab plus methotrexate arm), 325 significantly expressed GO terms were identified; in the purple module (tocilizumab arm) 304 GO terms were identified; and in the black module (methotrexate arm) 560 GO terms were identifies. The top five most significantly overrepresented GO terms (excluding terms with only one DEG) within the treatment arms are shown in Fig. 3. The most significant GO term in the tocilizumab plus methotrexate, tocilizumab, and methotrexate arms were “nuclear-transcribed mRNA catabolic process” (*p* < 1.00^E–04^), “granulocyte migration” (*p* = 2.70^E–04^), and “response to bacterium” (*p* = 1.92^E–07^), respectively. In addition, pathway analyses were performed using the KEGG database; “ribosome” was the only significant (*p* < 1.00^E–04^) pathway in the tocilizumab plus methotrexate arm; in the tocilizumab arm, no significant pathways were identified, and in the methotrexate arm “p53 signalling” (*p* = 8.44^E–06^) and “JAK-STAT signalling” (*p* = 2.22^E–04^) were significant pathways.

### Signature genes

**Fig. 4:**
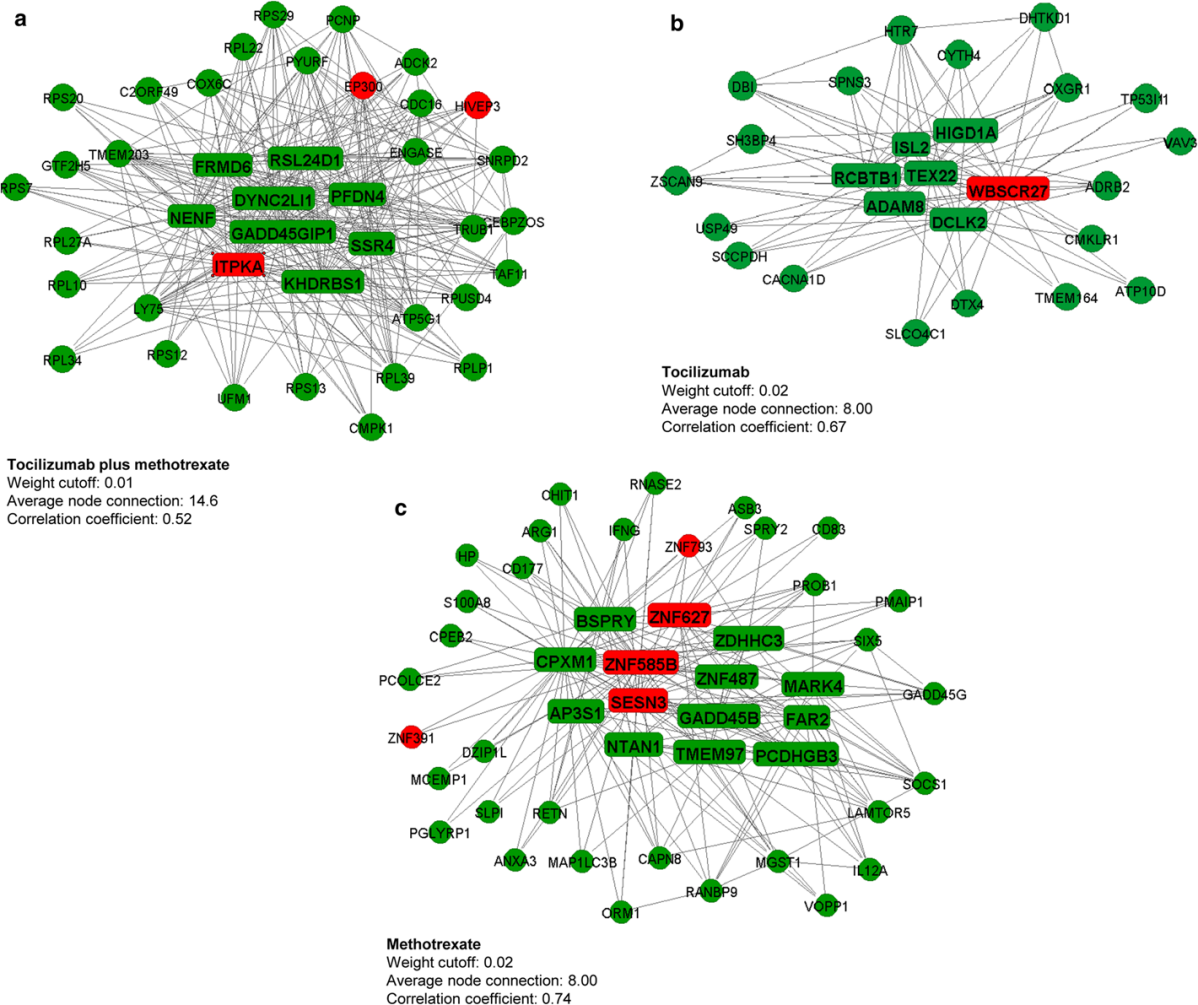
Network visualization of the interaction of gene co-expression within the tocilizumab plus methotrexate (**a**, salmon module), tocilizumab (**b**, purple module), and methotrexate (**c**, black module). Upregulated genes are expressed in *green* nodes and downregulated genes in *red* nodes. The *rounded* rectangular nodes display the highest co-expressed genes within the module (signature genes)

The interaction between genes in the modules selected for pathway analyses (tocilizumab plus methotrexate arm, salmon module; tocilizumab arm, purple module; methotrexate arm, black module) is shown in Fig. 4. Signature genes are visualized in the rounded rectangular nodes and genes with a lower connectivity are presented as round nodes. Across the three treatment arms, the large majority of the genes included in the modules were upregulated (green nodes) in those achieving sDFR. In the tocilizumab plus methotrexate arm, genes within the salmon module correlated best and, to select the most important genes, we applied a stricter cut-off. However, even when using a stricter cut-off, the average correlation between genes was the lowest (PCC 0.52) in this treatment arm when compared to the tocilizumab (PCC 0.67) and methotrexate (PCC 0.74) arms. Genes showing ≥10 connections within the modules were labelled as signature genes, except for the tocilizumab plus methotrexate arm, where genes were considered as important when showing ≥20 connections. In total, 9 (8 upregulated, 1 downregulated), 7 (6 upregulated, 1 downregulated), and 14 (11 upregulated, 3 downregulated) signature genes were identified in the tocilizumab plus methotrexate, tocilizumab, and methotrexate strategy arms, respectively.

## Discussion

In the present study, using analysis of networks of differentially co-expressed genes, several biological pathways were identified that were associated with achieving sDFR in DMARD-naive patients with early RA after initiating tocilizumab plus methotrexate-, tocilizumab-, or methotrexate-based treatment strategies. Across the three treatment arms, we found different networks, implying that there are several predisposing genes for achieving sDFR and, depending on the treatment strategy selected, patients have a higher chance of achieving sDFR if these are up- or downregulated. Some genes in particular that are included in the networks may play an important role as they are characterized by a high connectivity with other (signature) genes and therefore have the strongest influence on the pathways found relevant.

In the tocilizumab plus methotrexate strategy arm, the most important GO term was “nuclear-transcribed mRNA catabolic process, nonsense-mediated decay”, which is a pathway important in altering gene expression by degrading mRNA when converting amino acid-specific codons into premature stop codons (i.e. early termination of translation into proteins) [37]. After the genetic information from DNA is copied into RNA molecules during transcription, mRNA is decoded by ribosomes in the cytoplasm and is translated, using transfer RNA, into a specific sequence of amino acids (i.e. proteins). Translation starts with a start codon, which is the first codon of a mRNA transcript translated by a ribosome, and ends with a stop codon. Mutations, also known as single-nucleotide polymorphisms (SNPs), can occur in the sequence of nucleotide bases in DNA resulting in a different protein product. SNPs may not only increase the susceptibility to certain diseases but are also responsible for the differences in treatment response because of genetic variations. Other significant GO terms within this treatment arm are also related to processes involved in translation (GO:0006412) of mRNA by ribosomes (GO:0005840, GO:0003735) and is in accordance with the only identified significant KEGG pathway (“Ribosome”). Thus, it seems that pathways important for achieving sDFR in patients treated with tocilizumab plus methotrexate are in general related to processes involved with protein synthesis, especially during translation from mRNA into a sequence of amino acids. In the tocilizumab strategy arm, the most important GO terms in the purple module were related with migration of white blood cells (granulocytes and myeloid leukocytes) as a response to inflammation or to an external stimulus (i.e. leukocyte chemotaxis). Furthermore, G-protein-coupled receptor (GPCR) activity and signalling pathway (GO:0008227, GO:0007187) were significant GO terms within this module. GPCRs constitute a superfamily of receptor proteins that are involved in a many (patho)physiological processes and an important role of these receptors is to regulate the immune system and inflammation by activating internal signal transduction across the cell membrane, which eventually leads to cellular responses [38]. It is estimated that approximately two-third of all available drugs targets the GPCRs; the most commonly used in RA is methotrexate that works via a specific subtype of adenosine receptors, which is part of the large GPCR family [38]. The exact mechanism and the downstream effect ultimately leading to cellular responses of many GPCRs are still not fully elucidated, but they have been shown to be effective in several auto-immune diseases in reducing disease symptoms when specific receptors are targeted. Although it is known that tocilizumab inhibits the binding of IL-6 to its receptor, which belongs to the family of the type I cytokine receptors and thus does not seem to directly affect GPCRs, it might have an indirect effect on certain GPCRs. Further research is, however, required to determine and understand the potential effect of tocilizumab on these receptors. In the methotrexate arm, all selected GO terms were related to a response to some kind of bacterium or biotic (i.e. biological material) stimulus resulting in a change of state or activity of a cell. When analysing the GO ancestor chart, all terms were direct subtypes of “response to stimulus” (GO:0050896) indicating that the genes included in the black module may play a direct role in the response to therapy. However, the exact mechanisms of methotrexate in RA remain partially unclear, but multiple processes seem to play a role in suppressing disease activity. Although it is known, for example, that methotrexate inhibits cell proliferation and apoptosis, it was also found to inhibit cytokine production by inducing T-cell and B-cell activation [39]. Although many ex vivo and in vitro studies have been performed, the exact mechanism of action of cytokine level reduction by methotrexate remains largely not understood [39]. An important function of methotrexate may be the inhibiting effect on “Janus Kinase Signal Transducer and Activator of Transcription (JAK-STAT)” signalling, which was also a significantly expressed KEGG pathway in the black module (methotrexate arm). JAKs are enzymes transducing signals of cytokine receptors and, by inhibiting one or more enzymes, they prevent activation of cytokines important for the immune response [40]. A previous study has shown that methotrexate suppresses the JAK-STAT pathway; this could be an important mechanism for reducing RA disease activity [41]. Another significant KEGG pathway in the methotrexate arm was associated with signalling of the tumour protein p53 (“p53 signalling pathway”), which functions as a tumour suppressor and is thus not directly related to the pathogenesis of RA. This protein was, however, previously found to be involved with methotrexate-resistance in osteosarcoma and, therefore, it seems that genes important in the p53 pathway may be related to the response to methotrexate therapy in general [42].

In the present study, we analysed two different clusters of cells, CD4^+^ T-helper cells and CD14^+^ monocytes, for transcriptional profiling as both are important in modulating the immune response but have different functions and could thus have different pathways involved. T-helper cells are essential in the adaptive immunity by producing cytokines when pathogens are presented by monocytes and are therefore regulators of the immune response. Besides recruiting lymphocytes (antigen presentation), monocytes also perform phagocytosis after becoming macrophages, and are thus also important for the removal of pathogens. Although both clusters of cells are important within RA, when performing gene co-expression network analyses, no relevant modules were found in the CD14^+^ cells and only transcripts sequenced from CD4^+^ cells seems to play a role in achieving sDFR in DMARD-naive patients after initiating a tocilizumab- or methotrexate-based strategy. Other cells, such as B lymphocytes, also play an important role in auto-immune diseases and thus may contribute to the understanding of genetic variations within RA patients. However, these cells were not analysed in this study as they were not isolated because of the limited quantity of blood that had been collected at baseline and cost-related considerations.

## Conclusions

In conclusion, by performing network analyses of co-expressed genes we have identified several pathways related to achieving sDFR within DMARD-naive early RA patients after initiation of tocilizumab plus methotrexate-, tocilizumab-, or methotrexate-based treatment strategies. Within each of these three strategy arms, we found different biological pathways implicating that are different networks of predisposing genes related to achieving sDFR which are dependent on the type of treatment strategy selected. In addition, we identified several important genes within the networks that could potentially act as prognostic biomarkers for RA.

## Additional files

Additional file 1: Baseline characteristics of the patients included in the analyses. (DOCX 17 kb)

Additional file 2: Description of the differentially co-expressed genes in the salmon module (tocilizumab plus methotrexate arm), the purple module (tocilizumab arm), and the black module (methotrexate arm). (DOCX 22 kb)

Additional file 3: Correlations and corresponding *P* values between the modules and achieving sustained drug-free remission within sequenced CD14^+^ cells. (DOCX 25 kb)

## Abbreviations

CD: Cluster of differentiation
CRP: C-reactive protein
DAS28: Disease activity score assessing 28 joints
DEG: Differentially expressed gene
DMARD: Disease-modifying anti-rheumatic drug
FACS: Fluorescence-activated cell sorting
GO: Gene Ontology
GPCR: G-protein-coupled receptor
HLA: Human leucocyte antigen
IL: Interleukin
IQR: Interquartile range
JAK-STAT: Janus kinase signal transducer and activator of transcription
KEGG: Kyoto Encyclopedia of Genes and Genomes
mRNA: Messenger RNA
PAD14: Peptidyl arginine deiminase, type 14
PCA: Principal component analysis
PCC: Pearson’s correlation coefficient
PTPN22: Protein tyrosine phosphatase, non-receptor type 22
RA: Rheumatoid arthritis
RIN: RNA integrity number
RNA: Ribonucleic acid
RNA-seq: RNA sequencing
SD: Standard deviation
sDFR: Sustained drug-free remission
SNPs: Single-nucleotide polymorphisms
WGCNA: Weighted gene co-expression network analysis
