# Supplementary material for: Identification of differential co-expressed gene networks in early rheumatoid arthritis achieving sustained drug-free remission after treatment with a tocilizumab-based or methotrexate-based strategy
Source: Arthritis Res Ther. 2017 Jul 20;19:170. doi: 10.1186/s13075-017-1378-x (PMC5520225; doi:10.1186/s13075-017-1378-x)
Supplement: Supplementary file 1 — Baseline characteristics of the patients included in the analyses. (DOCX 17 kb) [file 13075_2017_1378_MOESM1_ESM.docx]

| **Additional file S1: Table S1** | | | | | | | | |
| --- | --- | --- | --- | --- | --- | --- | --- | --- |
| **Baseline characteristics of the patients included in the analyses.** | | | | | | | | |
|  | Tocilizumab plus methotrexate | |  | Tocilizumab | |  | Methotrexate | |
|  | sDFR  (n=14) | No sDFR  (n=5) |  | sDFR  (n=13) | No sDFR  (n=11) |  | sDFR  (n=10) | No sDFR  (n=7) |
| Female gender, n (%) | 6 (43) | 4 (80) |  | 9 (69) | 8 (73) |  | 8 (80) | 6 (86) |
| Age (years) | 53 (16) | 64 (10) |  | 58 (14) | 51 (13) |  | 50 (14) | 46 (17) |
| BMI (kg/m^2^) | 25 (4) | 27 (4) |  | 25 (2) | 45 (5) |  | 29 (4) | 26 (3) |
| Caucasian, n (%) | 13 (93) | 4 (80) |  | 13 (100) | 10 (91) |  | 10 (100) | 7 (100) |
| Current smokers, n (%) | 3 (21) | 1 (20) |  | 2 (15) | 3 (27) |  | 1 (10) | 1 (14) |
| Symptom duration (days), median (IQR) | 22 (21-40) | 19 (14-55) |  | 24 (18-39) | 21 (16-25) |  | 30 (13-40) | 31 (20-45) |
| RF positive, n (%) | 5 (34) | 3 (60) |  | 8 (62) | 6 (55) |  | 9 (90) | 5 (71) |
| Anti-CCP positive, n (%) | 5 (34) | 3 (60) |  | 8 (62) | 7 (64) |  | 7 (70) | 6 (86) |
| CRP (mg/L), median (IQR) ^¶^ | 5 (2-13) | 5 (4-9) |  | 15 (4-27) | 14 (4-30) |  | 11 (5-18) | 5 (4-12) |
| ESR (mm/h), median (IQR) ^¶^ | 18 (12-39) | 25 (23-29) |  | 26 (14-28) | 20 (9-39) |  | 25 (13-47) | 16 (13-25) |
| DAS28 (range 0-9.4, 9.4=maximum) | 4.7 (1.2) | 5.1 (0.9) |  | 5.0 (1.1) | 5.3 (1.3) |  | 4.6 (1.2) | 4.8 (0.9) |
| HAQ (range 0-3, 3=worst function) | 0.8 (0.5) | 1.5 (0.9) |  | 1.0 (0.6) | 1.4 (0.7) |  | 0.9 (0.6) | 1.0 (0.5) |
| Sharp/van der Heijde score, median (IQR) | 0 (0-0) | 0 (0-0) |  | 0 (0-3) | 0 (0-2) |  | 0 (0-1) | 0 (0-0) |
| Continuous data presented as mean (SD) unless otherwise indicated. No significant differences within the strategy arms between the subgroups (DFR *vs* no sDFR). SD = standard deviation; IQR = interquartile range; sDFR = sustained drug-free remission; BMI = body mass index; RF = rheumatoid factor; CCP = cyclic citrullinated peptide; CRP = c-reactive protein; ESR = erythrocyte sedimentation rate; DAS28 = disease activity assessing 28 joints; HAQ = health assessment questionnaire. | | | | | | | | |
