# Supplementary material for: Identification of differential co-expressed gene networks in early rheumatoid arthritis achieving sustained drug-free remission after treatment with a tocilizumab-based or methotrexate-based strategy
Source: Arthritis Res Ther. 2017 Jul 20;19:170. doi: 10.1186/s13075-017-1378-x (PMC5520225; doi:10.1186/s13075-017-1378-x)
Supplement: Supplementary file 2 — Description of the differentially co-expressed genes in the salmon module (tocilizumab plus methotrexate arm), the purple module (tocilizumab arm), and the black module (methotrexate arm). (DOCX 22 kb) [file 13075_2017_1378_MOESM2_ESM.docx]

| **Additional file 2: Table S2** | | |
| --- | --- | --- |
| **Description of the differentially co-expressed genes in the salmon module (tocilizumab plus methotrexate arm), the purple module (tocilizumab arm), and the black module (methotrexate arm).** | | |
| **Abbreviation** | **ENSEMBLE ID** | **Description** |
| **Tocilizumab plus methotrexate** | | |
| ADCK2 | ENSG00000133597 | Aarf Domain Containing Kinase 2 |
| ATP5G1 | ENSG00000159199 | ATP Synthase, H+ Transporting, Mitochondrial Fo Complex Subunit C1 (Subunit 9) |
| C2ORF49 | ENSG00000135974 | Chromosome 2 Open Reading Frame 49 |
| CDC16 | ENSG00000130177 | Cell Division Cycle 16 |
| CMPK1 | ENSG00000162368 | Cytidine/Uridine Monophosphate Kinase 1 |
| COX6C | ENSG00000164919 | Cytochrome C Oxidase Subunit 6C |
| DYNC2LI1 | ENSG00000138036 | Dynein Cytoplasmic 2 Light Intermediate Chain 1 |
| CEBPZOS | ENSG00000218739 | CEBPZ Opposite Strand |
| ENGASE | ENSG00000167280 | Endo-Beta-N-Acetylglucosaminidase |
| EP300 | ENSG00000100393 | E1A Binding Protein P300 |
| FRMD6 | ENSG00000139926 | FERM Domain Containing 6 |
| GADD45GIP1 | ENSG00000179271 | Growth Arrest and DNA-Damaga-Inducible, Gamma Interacting Protein 1 |
| GTF2H5 | ENSG00000272047 | General Transcription Factor IIH Subunit 5 |
| HIVEP3 | ENSG00000127124 | Human Immunodeficiency Virus Type I Enhancer Binding Protein 3 |
| ITPKA | ENSG00000137825 | Inositol-Triphosphate 3-Kinase A |
| KHDRBS1 | ENSG00000121774 | KH RNA Binding Containing, Signal Transduction Associated 1 |
| LY75 | ENSG00000054219 | Lymphocyte Antigen 75 |
| NENF | ENSG00000117691 | Neudesin Neurotrophic Factor |
| PCNP | ENSG00000081154 | PEST Proteolytic Signal Containing Nuclear Protein |
| PFDN4 | ENSG00000101132 | Prefoldin Subunit 4 |
| PYURF | ENSG00000145337 | PIGY Upstream Reading Frame |
| RPL10 | ENSG00000147403 | Ribosomal Protein L10 |
| RPL22 | ENSG00000116251 | Ribosomal Protein L22 |
| RPL27A | ENSG00000166441 | Ribosomal Protein L27a |
| RPL34 | ENSG00000109475 | Ribosomal Protein L34 |
| RPL39 | ENSG00000198918 | Ribosomal Protein L39 |
| RPLP1 | ENSG00000137818 | Ribosomal Protein Lateral Stalk Subunit P1 |
| RPS12 | ENSG00000112306 | Ribosomal Protein S12 |
| RPS13 | ENSG00000110700 | Ribosomal Protein S13 |
| RPS20 | ENSG00000008988 | Ribosomal Protein S20 |
| RPS29 | ENSG00000213741 | Ribosomal Protein S29 |
| RPS7 | ENSG00000171863 | Ribosomal Protein S7 |
| RPUSD4 | ENSG00000165526 | RNA Pseudouridylate Synthase Domain Containing 4 |
| RSL24D1 | ENSG00000137876 | Ribosomal L24 Domain Containing 1 |
| SNRPD2 | ENSG00000125743 | Small Nuclear Ribonucleoprotein D2 Polypeptide |
| SSR4 | ENSG00000180879 | Signal Sequence Receptor Subunit 4 |
| TAF11 | ENSG00000064995 | TATA-Box Binding Protein Associated Factor 11 |
| TMEM203 | ENSG00000187713 | Transmembrane Protein 203 |
| TRUB1 | ENSG00000165832 | TruB Pseudouridine Synthase Family Member 1 |
| UFM1 | ENSG00000120686 | Ubiquitin Fold Modifier 1 |

| ***Additional file 1 (continued)*** | | |
| --- | --- | --- |
| **Abbreviation** | **ENSEMBLE ID** | **Description** |
| **Tocilizumab** | | |
| ADAM8 | ENSG00000151651 | ADAM Metallopeptide Domain 8 |
| ADRB2 | ENSG00000169252 | Adrenoceptor Beta 2 |
| ATP10D | ENSG00000145246 | ATPase Phospholipid Transporting 10D (Putative) |
| CACNA1D | ENSG00000157388 | Calcium Voltage-Gated Channel Subunit Alpha 1D |
| CMKLR1 | ENSG00000174600 | Chemerin Chemokine-Like Receptor 1 |
| CYTH4 | ENSG00000100055 | Cytohesin 4 |
| DBI | ENSG00000155368 | Diazepam Binding Inhibitor, Acyl-CoA Binding Protein |
| DCLK2 | ENSG00000170390 | Doublecortin Like Kinase 2 |
| DHTKD1 | ENSG00000181192 | Dehydrogenase E1 and Transketolase Domain Containing 1 |
| DTX4 | ENSG00000110042 | Deltex E3 Ubiquitin Ligase 4 |
| HIGD1A | ENSG00000181061 | HIG1 Hypoxia Inducible Domain Family Member 1A |
| HTR7 | ENSG00000148680 | 5-Hydroxytryptamine Receptor 7 |
| ISL2 | ENSG00000159556 | ISL LIM Homeobox 2 |
| OXGR1 | ENSG00000165621 | Oxoglutarate Receptor 1 |
| RCBTB1 | ENSG00000136144 | RCC1 and BTB Domain Containing Protein 1 |
| SCCPDH | ENSG00000143653 | Saccharopine Dehydrogenase (Putative) |
| SH3BP4 | ENSG00000130147 | SH3 Domain Binding Protein 4 |
| SLCO4C1 | ENSG00000173930 | Solute Carrier Organic Anion Transporter Family Member 4C1 |
| SPNS3 | ENSG00000182557 | Sphingolipid Transporter 3 (Putative) |
| TEX22 | ENSG00000226174 | Testis Expressed 22 |
| TMEM164 | ENSG00000157600 | Transmembrane Protein 164 |
| TP53I11 | ENSG00000175274 | Tumor Protein P53 Inducible Protein 11 |
| USP49 | ENSG00000164663 | Ubiquitin Specific Peptidase 49 |
| VAV3 | ENSG00000134215 | Vav Guanine Nucleotide Exchange Factor 3 |
| WBSCR27 | ENSG00000165171 | Williams Beuren Syndrome Chromosome Region 27 |
| ZSCAN9 | ENSG00000137185 | Zinc Finger and SCAN Domain Containing 9 |
| **Methotrexate** | | |
| ARG1 | ENSG00000118520 | Arginase 1 |
| ASB3 | ENSG00000115239 | Ankyrin Repeat and SOCS Box Containing 3 |
| BSPRY | ENSG00000119411 | B-Box and SPRY Domain Containing |
| CAPN8 | ENSG00000203697 | Calpain 8 |
| CD177 | ENSG00000204936 | CD177 Molecule |
| CD83 | ENSG00000112149 | CD83 Molecule |
| CHIT1 | ENSG00000133063 | Chitinase 1 |
| CPEB2 | ENSG00000137449 | Cytoplasmica Polyadenylation Element Binding Protein 2 |
| CPXM1 | ENSG00000088882 | Carboxypeptidase X, M14 Family Member 1 |
| DZIP1L | ENSG00000158163 | DAZ Interacting Zinc Finger Protein 1 Like |
| FAR2 | ENSG00000064763 | Fatty Acyl-CoA Reductase 2 |
| GADD45B | ENSG00000099860 | Growth Arrest and DNA Damage Inducible Beta |
| GADD45G | ENSG00000130222 | Growth Arrest and DNA Damage Inducible Gamma |
| HP | ENSG00000257017 | Haptoglobin |
| IFNG | ENSG00000111537 | Interferon Gamma |

| ***Additional file 1 (continued)*** | | |
| --- | --- | --- |
| **Abbreviation** | **ENSEMBLE ID** | **Description** |
| IL12A | ENSG00000168811 | Interleukin 12A |
| LAMTOR5 | ENSG00000134248 | Late Endosomal/Lysosomal Adaptor, MAKP and MTOR Activator 5 |
| MAP1LC3B | ENSG00000140941 | Microtubule Associated Protein 1 Light Chain 3 Beta |
| MARK4 | ENSG00000007047 | Microtubule Affinity Regulating Kinase 4 |
| MCEMP1 | ENSG00000183019 | Mast Cell Expressed Membrane Protein 1 |
| MGST1 | ENSG00000008394 | Microsomal Glutathione S-Transferase 1 |
| NTAN1 | ENSG00000157045 | N-Terminal Asparagine Amidase |
| ORM1 | ENSG00000229314 | Orosomucoid 1 |
| PCDHGB3 | ENSG00000262209 | Protocadherin Gamma Subfamily B, 3 |
| PCOLCE2 | ENSG00000163710 | Procollagen C-Endopeptidase Enhancer 2 |
| PGLYRP1 | ENSG00000008438 | Peptidoglycan Recognition Protein 1 |
| PMAIP1 | ENSG00000141682 | Phorbol-12-Myristate-13-Acetate-Induced Protein 1 |
| PROB1 | ENSG00000228672 | Proline Rich Basic Protein 1 |
| RANBP9 | ENSG00000010017 | RAN Binding Protein 9 |
| RETN | ENSG00000104918 | Resistin |
| RNASE2 | ENSG00000169385 | Ribonuclease A Family Member 2 |
| S100A8 | ENSG00000143546 | S100 Calcium Binding Protein A8 |
| SESN3 | ENSG00000149212 | Sestrin 3 |
| SIX5 | ENSG00000177045 | SIX Homeobox 5 |
| SLPI | ENSG00000124107 | Secretory Leukocyte Peptidase Inhibitor |
| SOCS1 | ENSG00000185338 | Suppressor of Cytokine Signalling 1 |
| SPRY2 | ENSG00000136158 | Sprouty RTK Signalling Antagonist 2 |
| TMEM97 | ENSG00000109084 | Transmembrane Protein 97 |
| VOPP1 | ENSG00000154978 | Vesicular, Overexpressed in Cancer, Prosurvival Protein 1 |
| ZDHHC3 | ENSG00000163812 | Zinc Finger DHHC-Type Containing 3 |
| ZNF391 | ENSG00000124613 | Zinc Finger Protein 391 |
| ZNF487 | ENSG00000243660 | Zinc Finger Protein 487 |
| ZNF585B | ENSG00000245680 | Zinc Finger Protein 585B |
| ZNF627 | ENSG00000198551 | Zinc Finger Protein 627 |
| ZNF793 | ENSG00000188227 | Zinc Finger Protein 793 |
