# Supplementary material for: Identification of differential co-expressed gene networks in early rheumatoid arthritis achieving sustained drug-free remission after treatment with a tocilizumab-based or methotrexate-based strategy
Source: Arthritis Res Ther. 2017 Jul 20;19:170. doi: 10.1186/s13075-017-1378-x (PMC5520225; doi:10.1186/s13075-017-1378-x)
Supplement: Supplementary file 3 — Correlations and corresponding P values between the modules and achieving sustained drug-free remission within sequenced CD14+ cells. (DOCX 25 kb) [file 13075_2017_1378_MOESM3_ESM.docx]

| **Additional file S3: Table S2** | | | | | |
| --- | --- | --- | --- | --- | --- |
| **Correlations and corresponding *P*-values between modules and achieving sustained-drug free remission within sequenced CD14^+^ cells.** | | | | | |
|  |  | **Module** | **Size^†^** | **Correlation** | ***P*** |
| Tocilizumab plus methotrexate | 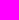 | Magenta | 16 | 0.18 | NS |
|  | 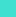 | Turquoise | 557 | 0.16 | NS |
|  | 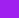 | Purple | 12 | 0.12 | NS |
|  | 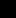 | Black | 20 | 0.12 | NS |
|  | 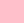 | Pink | 18 | 0.10 | NS |
|  | 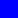 | Blue | 238 | 0.10 | NS |
|  | 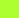 | Greenyellow | 11 | -0.04 | NS |
|  | 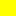 | Yellow | 64 | -0.09 | NS |
|  | 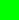 | Green | 56 | -0.29 | NS |
| Tocilizumab | 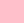 | Pink | 22 | 0.41 | 0.049 |
|  | 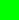 | Green | 52 | 0.37 | NS |
|  | 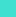 | Turquoise | 587 | 0.11 | NS |
|  | 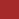 | Brown | 62 | -0.24 | NS |
|  | 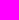 | Magenta | 21 | -0.24 | NS |
|  | 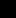 | Black | 23 | -0.37 | NS |
|  | 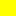 | Yellow | 53 | -0.38 | NS |
|  | 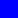 | Blue | 122 | -0.44 | 0.033 |
|  | 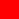 | Red | 38 | -0.51 | 0.011 |
| Methotrexate | 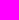 | Magenta | 21 | 0.42 | NS |
|  | 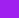 | Purple | 20 | 0.38 | NS |
|  | 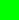 | Green | 87 | 0.35 | NS |
|  | 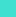 | Turquoise | 359 | 0.27 | NS |
|  | 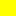 | Yellow | 58 | 0.25 | NS |
|  | 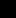 | Black | 27 | -0.02 | NS |
|  | 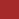 | Brown | 395 | -0.41 | NS |
|  | 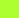 | Greenyellow | 17 | -0.48 | NS |
|  | 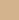 | Tan | 15 | -0.56 | 0.019 |
| The module eigengene, which is the first principal component of the expression matrix within the module of interest, was considered as the average gene expression profile and was used to test the correlation between each module and achieving sustained drug-free remission. NS = not significant. **^†^** The minimal number of genes per module was set at 10, except for the tocilizumab arm (n=20). | | | | | |
